# Advancement of Nanobiomaterials to Deliver Natural Compounds for Tissue Engineering Applications

**DOI:** 10.3390/ijms21186752

**Published:** 2020-09-15

**Authors:** Sathish Sundar Dhilip Kumar, Heidi Abrahamse

**Affiliations:** Laser Research Centre, Faculty of Health Sciences, University of Johannesburg, Johannesburg 2028, South Africa; sathishd@uj.ac.za

**Keywords:** nanocarrier, natural compounds, tissue engineering, scaffolds, biomaterials

## Abstract

Recent advancement in nanotechnology has provided a wide range of benefits in the biological sciences, especially in the field of tissue engineering and wound healing. Nanotechnology provides an easy process for designing nanocarrier-based biomaterials for the purpose and specific needs of tissue engineering applications. Naturally available medicinal compounds have unique clinical benefits, which can be incorporated into nanobiomaterials and enhance their applications in tissue engineering. The choice of using natural compounds in tissue engineering improves treatment modalities and can deal with side effects associated with synthetic drugs. In this review article, we focus on advances in the use of nanobiomaterials to deliver naturally available medicinal compounds for tissue engineering application, including the types of biomaterials, the potential role of nanocarriers, and the various effects of naturally available medicinal compounds incorporated scaffolds in tissue engineering.

## 1. Introduction

In medicine, nanotechnology can develop effective treatment modalities and overcome the challenges associated with the diagnosis, prevention, and treatment of various diseases [1,2]. It is extensively used in various perspectives on nanomedicine, including drug delivery (nanoscale delivery vehicles), in vitro diagnostics/detection (nano-based sensors), in vivo imaging (targeting imaging nanoprobes), therapy techniques (metal-based nanoparticles in hypothermia/antimicrobial agent), biomaterials (biocompatible medical implants), and tissue engineering (scaffolds which mimic extracellular matrix (ECM)). Recent advances in nanotechnology provide an easy process for designing nanocarrier-based scaffolds for the purpose and specific use of tissue engineering applications [3] such as the sustained delivery of drugs, bioactive molecules and angiogenic factors [4]. Tissue engineering is an interdisciplinary field based on the principles from life sciences and engineering to restore, improve and maintain tissue function [5]. Tissue engineering scaffolds have certain requirements such as physical, chemical, and mechanical properties to enhance cell diffusion and three-dimensional (3D) tissue formation [6]. The biocompatibility, biodegradability, and mechanical properties of scaffolds play an important role in tissue regeneration, and provide adequate support to cells. Biocompatible scaffolds allow the cells to function normally by enhancing their surface adhering and migration properties. The primary purpose of using scaffolds in tissue engineering is to allow cells to build their ECM and to be completely biodegradable without activating any immune reactions in our body. The by-products of degraded scaffold should be non-toxic and can be removed from our body without causing any adverse effects. Eventually, the scaffolding material must have good mechanical properties, including tensile strength and compressive stiffness, to maintain their integrity during implantation [7].

Biomaterial-based scaffolds or implants have played an important role in increasing support for cell growth in tissue engineering and regenerative medicine. The biomaterial must be fabricated based on the structural, biochemical, and biological requirements for tissue engineering applications [8]. Currently, research on the biomaterial-based delivery of natural medicinal compounds and their scientific implications in tissue engineering applications has attracted global attention and the World Health Organization (WHO) reports that about 80% of the world’s population relies on traditional medicine. It is well documented that plants have been identified as a major source of natural medicinal compounds and that plant-based compounds (phytochemicals) are considered to be highly effective in topical applications with reduced side effects [3,9,10]. Some natural medicinal compounds derived from plant sources and their active ingredients are listed here including turmeric (curcumin), garlic (allicin), *Aloe vera* (Acemannan), ginger (gingerol), soy (isoflavones), green tea (epigallocatechin-gallate) played a functional role with positive outcomes in bone tissue engineering [11].

In this review article, we focus primarily on the progress in using nanocarriers to deliver naturally available medicinal compounds for tissue engineering application. Accordingly, this review article is organized into five different sections. In Section 1, an introduction to the study is briefly explained. In Section 2, the use of different biomaterials and their potential role in tissue engineering applications is presented. In Section 3, the different types of nanocarriers available in tissue engineering applications and their potential benefits are discussed in detail. In Section 4, the effects of natural compounds incorporated scaffold in tissue engineering applications are described, as well as various methods to fabricate the scaffolds. In Section 5, some conclusions are presented about the future perspectives of this work.

## 2. Biomaterials for Tissue Engineering

Natural and synthetic polymers have been identified and are widely used as a biomaterial for tissue engineering applications because of their high level of cellular compatibility (non-toxic), simple design, preparation, structural stability, enhanced adhesion, biodegradability, and interestingly, their biomimetic properties [12].

### 2.1. Natural Polymer-Based Biomaterials

The commonly used, natural, polymer-based biomaterials are collagen, silk, gelatin, keratin, chitosan, hyaluronan, starch, pullulan, cellulose, alginate, and chondroitin. Collagen is a Food and Drug Administration (FDA)-approved material for a variety of biomedical applications, including wound dressing and artificial skin [13]. Collagen derived from animal origin retains the risks of immune responses and interestingly, the use of other natural biomaterials (e.g., silk fibroin) can overcome the side effects associated with these collagens [14]. It can act as a biomaterial in tissue engineering from two different methods, the first being a decellularized form of collagen and the second forming a scaffold by mixing collagen with other biomolecules [15]. Collagen-based biomaterials serve as an excellent vehicle to deliver cellular components and bioactive molecules for myocardial repair and regeneration [16]. Stem-cell-specific antibodies such as anti-Sca-1 loaded collagen scaffold were successfully synthesized to target Sca-1-positive cells to promote myocardial regeneration in a mouse model. The anti-Sca-1 conjugated scaffold effectively enhances the regeneration of cardiomyocytes at the site of injury in the myocardium [17]. Yoon et al. (2020) recently studied the role and application of type I collagen-based biomaterial (Insuregraf^®^) as a skin graft and suggested that this material is clinically suitable for use as a dermal substitute in the treatment of burn wounds [18]. Boccafoschi et al. (2005) prepared reconstituted collagen films for vascular tissue engineering applications, and the cell culture study results revealed that the material supports cell spreading based on the observation of their cell adhesion and proliferation properties [19]. Improved mechanical properties and the bioactivity of biomimetic type I collagen and elastin-based meshes showed positive effects for the treatment of ventral hernia repair in the in vivo rat model [20].

In recent decades, the use of silk fibroin (SF) in tissue engineering has increased due to their simple fabrication process, excellent biocompatibility and biodegradability [21,22] and it can be used successfully as a biomaterial in various tissue engineering applications including musculoskeletal [23], bone [24], soft [25], cartilage [26], cardiac [27] and neural tissue engineering [28]. The tailoring properties of SF-based porous aerogels have demonstrated good cytocompatibility against human foreskin fibroblast cells and can be used for tissue engineering applications [29]. Bhardwaj et al. (2011) fabricated SF, chitosan-based scaffolds for cartilage tissue engineering applications, with the biochemical and mechanical properties of the scaffolds resulting in enhanced cell attachment and the accumulation of glycosaminoglycan and collagen on the synthesized scaffolds [30]. The functional properties of nanocomposite based on hydroxyapatite and SF enhanced the bioactivity of bone growth and the content of SF in the nanocomposite played a significant role in cell proliferation, biodegradation and biomineralization for bone tissue engineering applications [31]. The surface morphology and biocompatibility of SF and cobalt ferrite nanoparticle compounds played an important role in the cell viability and proliferation rate of MC3T3-E1 pre-osteoblast cells and were suitable for bone tissue engineering applications [32]. Growth factor incorporated SF-based scaffolds has limitations in the release profile and rapid loss of the loaded material. The growth factor gene sequences functionalized silk fibers secreted from transgenic silkworms and sustained the presence of growth factors on the scaffolds, and it significantly enhanced cell proliferation and wound closure [33].

Gelatin is a biocompatible and biodegradable natural polymer which is derived from collagen hydrolysis, and it has numerous applications as a scaffold in tissue engineering and carrier molecule in drug delivery [34,35]. There are some disadvantages of using gelatin in tissue engineering applications which include poor mechanical and thermal properties, it can be easily overcome by making a composite material [36]. Some of the gelatin-based composite materials and their potential benefits as a scaffold material for tissue engineering applications are briefly discussed here. Nanocomposite fibers based on gelatin and cerium oxide nanoparticles have shown a positive effect in nerve tissue engineering and regenerative medicine [37]. Dual-nanofiber scaffolds based on polyurethane-gelatin and nylon 6-gelatin are made by electrospinning, which has a good tensile strength and wettability properties, and the porous structure of gelatin scaffolds promotes osteoblast cell attachment, migration, and proliferation [38]. Nooeaid et al. (2020) developed a biocompatible, multifunctional, highly porous tetracycline hydrochloride/polylactic acid/gelatin-based gel for use in soft-tissue engineering. The scaffold material exhibits excellent antibacterial activity against *Staphylococcus aureus* and *Escherichia coli* bacterial strains and has shown cytocompatibility with human dermal fibroblast cells [39]. Sharifi et al. (2020) fabricated gelatin/chondroitin sulfate/polycaprolactone-based nanofibrous scaffolds and has shown better human mesenchymal stem cells (hMSCs) attachment and chondrogenesis differentiation for cartilage tissue engineering applications [40]. The ciprofloxacin-loaded three-dimensional porous phosphate glass-reinforced gelatin scaffolds for bone tissue engineering were synthesized; the scaffolds mimic ECM properties, and sustained release of ciprofloxacin was observed in the phosphate-buffered saline at 37 °C. Cell culture studies for these scaffolds were performed, and the results revealed good cell adhesion and proliferation with enhanced cell viability in osteoblast like MG-63 cells [41].

Keratin-based scaffold are biocompatible, the medicinal uses are well documented in the literature. The major sources of keratin are from hair, wool, horns, hooves and nails. It has shown some significant advantages in tissue engineering applications due to their intrinsic biological functions and the production of pure keratin fibers is a major physical limitation associated with keratin materials, which can be overcome by blending with synthetic and natural polymers [42]. The functional role of keratin-associated proteins and keratin intermediate filaments extracted from hair samples was prepared as a hybrid hydrogel with chitosan, which showed controllable mechanical properties for tissue engineering applications [43]. The porous composites of keratin-based three-dimensional scaffolds are prepared by keratin in combination with chitosan or gelatin without the use of any cross-linking agent, and they have shown better thermal stability, mechanical strength and biocompatibility, and enhanced proliferation rate of NIH3T3 fibroblasts cells and the newly synthesized healthy ECM compared to pure keratin [44]. Oxygen-generating smart scaffolds are prepared using a mixture of human keratin, SF, gelatin, and calcium peroxide for use in urinary tract tissue engineering. The synthesized smart scaffold demonstrated good antibacterial activity against *Staphylococcus aureus* and *Escherichia coli,* which are commonly found in urinary tract infections. The implantation of this smart scaffold study was conducted in an in vivo rabbit model to treat urethral defect; it showed enhanced cell proliferation of autologous cells and prevented fistula formation due to its excellent antibacterial activities [45]. Dou et al. (2020) recently reported the use of sulfonated keratin- and polycaprolactone-based mats for vascular tissue engineering applications and the synthesized mats were shown to play a key role in enhancing endothelial cells growth with better blood and cytocompatibility [46].

Chitosan has been extensively studied in tissue engineering applications due to their surface hydrophilicity, biocompatibility, biodegradability, and significant biochemical properties [47]. The chitosan membranes have low mechanical resistance, being as stiff and brittle in nature which are considered as major disadvantage of using chitosan as a scaffold in tissue engineering. The mechanical properties are effectively improved by cross-linking with hydrophilic material such as polyethylene glycol [48,49]. An electrostatically immobilized heparin containing chitosan scaffolds stimulates osteoblast proliferation and demonstrates the enhanced cell viability and differentiation in MC3T3-E1 in vitro for bone tissue engineering applications [50]. In another study, an immobilized heparin containing chitosan scaffolds improved the stability and loading efficiency of the nerve growth factor and supported morphological development with enhanced cell attachment and cell proliferation of Schwann cell in vitro, and it may be prominently used in peripheral nerve regeneration [51]. The biomimetic vascular microenvironment was constructed with a combination of heparin, vascular-endothelial-growth-factor-loaded chitosan and a polycaprolactone-based 3D nanofibrous scaffold by electrospinning method, and enhanced endothelial cell proliferation and anticoagulation properties [52]. Gomes et al. (2017) synthesized chitosan, polycaprolactone, and gelatin-based hybrid scaffolds by electrospinning method, showing better physicochemical and biological properties for skin tissue engineering applications [53]. Wang et al. (2017) demonstrated the use of hydrophilic poly (3,4-ethylenedioxythiophene)-based chitosan and gelatin porous scaffold for neural tissue engineering applications. Electroactive biomaterial significantly improved the electrical conductivity, mechanical and thermal properties of the scaffolds. An in vitro cell culture study revealed that the synthesized electroactive biomaterial showed enhanced biocompatibility, cell adhesion, proliferation, gene expression, and protein levels in PC12 cells [54]. Biomimetic genipin cross-linked collagen and chitosan-based porous scaffolds were prepared, the addition of chitosan played an important role in cross-linking efficiency, and the degradation study showed that the addition of genipin enhances the biostability of the material. The cross-linked scaffold showed excellent biocompatibility against rabbit chondrocytes in vitro and was recommended for use in articular cartilage tissue engineering [55].

Hyaluronic acid (HA) is a biocompatible mucopolysaccharide, a type of glycosaminoglycan which is considered a major component of ECM [56]. The major disadvantage of HA in scaffold preparation is its low stability and rapid degradation; often, chemical modification and cross-linking is required to make this material useful in tissue engineering applications [57]. Li et al. (2020) recently fabricated the interpenetrating network scaffolds based on collagen, chondroitin sulfate and HA. HA-containing scaffolds significantly enhanced neurogenesis and may be considered for use in brain tissue engineering therapy [58]. Core-shell-structured nanofibers were fabricated using polyurethane, starch, and HA. The biological properties of HA enhanced the scaffold-cell attachment in the L929 mouse fibroblasts cell (in vitro) study, and the core-shell structural morphology of nanofibers showed a positive impact on wound healing rate (in vivo) compared to control [59]. The mechanical properties of HA-based cryogels were improved by adding the halloysite nanotubes. The hemocompatibility study results revealed that the non-hemolytic nature of this scaffold and the scaffold treated cells showed an improved cellular activity in different cell types [60]. The HA-based microfibrous scaffolds supported the complete formation of monolayer in HUVECs cells, and the co-culture of HUVECs with MSCs study showed blood vessel formation on the scaffolds [61]. The ionic bonding between the cationic chitosan and the anionic HA forms the polyelectrolyte complex, which demonstrated advanced physicochemical, mechanical and biological properties of the chitosan-HA-based scaffolds for many applications in tissue engineering [62]. Some of the studies based on chitosan-HA scaffolds and their positive outcomes on tissue engineering applications are listed here. Chitosan-HA-based scaffolds enhance cartilage ECM production [63], act as a hydrogel for cartilage tissue engineering [64], can be used in dental pulp regeneration [65], acts as an injectable material in tissue engineering [66], and can be applied in bone defects and various bone tissue engineering applications [67,68,69].

Pullulan is a non-toxic, edible biopolymer derived from different fungal strains, and surface-modified pullulan-based scaffolds have received considerable attention in tissue engineering applications. Although non-toxic in nature, pullulan has its own limitations in tissue engineering, i.e., a lack of adhesive properties that do not support cell proliferation and osteogenesis [70]. Bae et al. demonstrated the functional application of cell-encapsulated and surface-modified pullulan-based hydrogel, which also improved the mechanical and biological properties of the scaffold [71]. Amrita et al. (2015) reported that the successfully manufactured porous pullulan scaffold has a nano-hydroxyapatite-based deposition to overcome its surface adhesive limitations. Surface-modified pullulan and their enhanced osteoconductivity can be used successfully in bone tissue engineering [72]. ECM-mimetic, chemically cross-linked pullulan- and gelatin-based nanogels can be used as scaffolds in tissue engineering [73]. In bone tissue engineering applications, antibiotic cefuroxime axetil-loaded pullulan, poly(hydroxybutyrate-co-hydroxyvalerate) and polycaprolactone and diatom shell-based 3D scaffolds have been developed and their osteocompatibility has been studied in Saos-2 cells, which has shown enhanced cell viability, cell attachment and cell distribution [74]. An injectable cross-linked scaffold was fabricated using pullulan, dextran and nanocrystalline hydroxyapatite and evaluated in rat femoral condyle defects. Smaller size microbeads (300–500 µm in diameter) were successfully filled and promoted ingrowth in the bone defect site in favor of bone formation and mineralization [75]. The cellular response of a 3D-printed, pullulan-based hydrogel scaffold was evaluated using both HEK293 and mesenchymal stem cells. The scaffold material demonstrated excellent cell viability in both two-dimensional (2D) and 3D patterns and showed excellent adhesive properties on the scaffold coated with ECM fibronectin [76]. Biomimetic and injectable pullulan-HA with hydroxyapatite-based hydrogels were prepared using silane coupling agents, which act as a dermal filler for long-lasting durability, and cell culture studies showed improvements in cell adhesion in L-929 fibroblast cells [77]. Pullulan- and cellulose-based crosslinked scaffolds were prepared by the electrospinning method, which showed improved stability and mechanical properties by different physicochemical characterizations. A prolonged cell culture study (35 days) in Saos-2 cells revealed the cytocompatibility of scaffolds with adjustable thickness and structural integrity, which allowed the cells to adhere and proliferate within the material and can be used successfully as a potential scaffold for tissue engineering applications [78].

Chondroitin sulfate-based biomaterial has their benefits in tissue engineering applications such as naturally derived biomimetic and bioactive macromolecules. It is used in various tissue engineering applications due to its biocompatibility, biodegradability, and anionic properties [79]. Crosslinking treatment with other stable polymer is always required to overcome the low-stability issues associated with chondroitin sulfate [80]. Hybrid biomimetic nanofibrous scaffolds based on the mixture of gelatin, polyvinyl alcohol and chondroitin sulfate were fabricated through electrospinning process and showed excellent mechanical and biological properties for skin tissue engineering applications [81]. Cross-linked chitosan, natural hydroxyapatite, chondroitin sulfate, and amylopectin-based scaffolds showed an interconnected porous structure, good water retention ability and controlled biodegradability suitable for bone growth and bone tissue engineering applications. Cell culture studies showed enhanced cell attachment and proliferation compared to chitosan scaffolds in MG-63 cells [82]. The lower roughness and enhanced hydrophilicity properties of chondroitin sulfate immobilized nanofiber meshes made it an excellent substrate for human articular chondrocytes in cartilage tissue engineering applications [83]. Chondroitin sulfate-based biomaterial shows positive effects on the treatment of articular cartilage defects in the animal model and exhibits considerable anti-inflammatory effects [84]. Chondroitin sulfate-based biomaterial accelerated the epidermal regeneration process [85], promoted cardiomyocyte proliferation [86], periodontal tissue engineering [87] and skin tissue engineering [81], improved the clinical efficacy of islet transplantation [88], bone tissue engineering [89], bone defect healing [90] and bone regeneration [91].

### 2.2. Synthetic Polymer-Based Biomaterials

Synthetic polymer-based biomaterials used for tissue engineering applications and some of them are polylactic acid (PLA), polyglycolic acid (PGA), poly (lactic-co-glycolic acid) (PLGA) and polycaprolactone. Multifunctional scaffolds based on a combination of synthetic (PLA) and natural polymers have attracted interest in soft tissue engineering and this approach has significantly enhanced the mechanical and compressive properties of PLA-based scaffolds [39]. Modified bioactive surface scaffolds were manufactured using PLA and HA with enhanced biocompatibility and pro-angiogenic activity and can be successfully used for tissue engineering applications [92]. The porous PLA-, polycaprolactone- and HA-based scaffolds were prepared by indirect 3D-printing for bone tissue engineering, which has shown good cell viability, attachment and proliferation on MG63 osteoblast cells and enhanced the function of alkaline phosphatase (ALP) and osteoblast [93]. In Wistar rat studies, the implanted Mesenchymal-stem cell-loaded PLA scaffolds have shown positive healing effects in the scaffold material when compared to control in bone tissue engineering and bone regeneration [94]. A novel scaffold was developed using tumor necrosis factor-α and insulin-like, growth-factor-loaded polycaprolactone and PLA to treat acute liver failure. The results demonstrated cell cycle arrest inhibition in the G1 phase and anti-senescence mechanisms induced by scaffold material, and the senescence genes were downregulated in HepG2 cells in the RT-PCR study. Hepatocellular molecules were detected by the immunocytochemical staining method to confirm the differentiation of bone marrow stem cells into hepatocytes and the results showed an upregulated expression of hepatocellular molecules in a scaffold-treated group compared to the control group. A urea estimation study was performed to identify the functions of differentiated hepatocytes and expressions were relatively improved compared to the control [95].

PLGA is a biodegradable synthetic polymer, which showed numerous advantages in tissue engineering applications [96]. The lack of bioactivity is the major drawback associated with PLGA [97]. The addition of bioactive glass can significantly improve the biological properties of PLGA-based scaffolds [98]. The fiber tubes were prepared using PGA and coated with poly (L-lactic acid) (PLLA) and PLGA. The PLLA-bonded tubes showed an advantageous result in a larger compressive force study and the degradation assay compared to PLGA-bonded tubes. The rat study revealed that there were no structural changes in the PLLA-bonded tubes implanted during fibrovascular tissue ingrowth [99]. The cellular performance of the porous PGA scaffold was evaluated with human skin fibroblasts cells in vitro, which demonstrated the evident cell adherence and proliferating property of scaffolds, and the scaffold porous structure was could be covered by ECM due to their biocompatibility. The in vivo compatibility of the scaffolds was studied by subcutaneous implantation in Sprague-Dawley rat models. The histocompatibility of the scaffold was further confirmed by the distribution of new blood cells and the presence of collagenous fiber on the implanted scaffold [100]. Positively charged poly-L-lysine (PLL) modified porous PLGA microspheres were used for tissue engineering applications and showed strong interactions with the negatively charged cell membrane, allowing the MG63 human osteoblast-like cells to spread evenly over the surface of the microspheres [101]. Ong et al. (2018) demonstrated the use of a PLGA-based biodegradable microporous scaffold as a carrier for both hydrophobic (curcumin) and hydrophilic (gentamicin) nature drugs [102]. Curcumin-loaded, PLGA-embedded chitosan scaffolds were successfully studied for the treatment of chronic wounds. The porous structure of the scaffold supported cell growth and proliferation in Vero cells (in vitro), and the curcumin-loaded composite scaffold displayed more potent antibacterial property against *Staphylococcus aureus* than the plain scaffold [103]. The novel PLGA- and PLA-based hybrid nanofibers were prepared by electrospinning method, which helped achieve a sustained release of loaded thymosin beta-4. The biocompatibility of nanofibers tested against human-adipose-derived mesenchymal stem cells and the encapsulated thymosin beta-4 played a significant role in cell migration and tenogenic differentiation of cells in tendon regeneration [104]. Qodratnama et al. (2015) reported the use of lysozyme as a model protein to study the release behavior properties of the PLGA polymer for tissue engineering applications. The results demonstrated that the synthesized PLGA-based microparticle could be used to control protein release. The released bioactive molecules and the surface morphology of the PLGA microsphere may support cellular responses and provide positive effects on cell growth and differentiation [105]. The sustained release of vascular endothelial growth factor (VEGF) and the enhanced cell proliferation rate was observed from VEGF-loaded, PLGA-coated beta-tricalcium phosphate scaffold compared to the scaffold without VEGF, which can be used for bone regeneration applications. [106]. In another study, VEGF-loaded TCP and PLGA microsphere-based scaffold prepared for the treatment of craniofacial defects by 3D-printing technology [107]. Polycaprolactone-based scaffolds are gradually used in tissue engineering applications due to their non-toxic, mechanical and tissue-compatible properties. It can be degraded by the hydrolysis process under physiological conditions, but the rate of degradation is slower due to the presence of repeating five hydrophobic CH_2_ moieties [108]. Polycaprolactone is an FDA-approved biodegradable polymer used in bone tissue engineering and the addition of biocompatible nanoparticles significantly overcomes the bioactivity issues related to polycaprolactone [109]. The polycaprolactone-based hybrid scaffold has shown cytocompatibility in SaoS-2 cells with enhanced cell viability and cell density compared to the control. Hybrid composite materials, such as calcium-polyphosphate microparticles with polycaprolactone, play an important role in improving the surface morphology of polycaprolactone, and appear as a smooth surface, whereas rough surface control is observed. Cell density and cell morphology were further examined by scanning electron microscopy, and no cells were detected on the rough surface of the control, whereas a polycaprolactone-based hybrid material showed a clearly visible cell morphology of SaoS-2 cells [110].

The polyethylene glycol (PEG)-based composite nanofibers were prepared by the electrospinning method and this showed enhanced tensile strength and cell adherence properties compared to cellulose acetate butyrate (CAB) alone. Here, the hydrophilicity nature of polyethylene glycol (PEG) plays a key role in reducing the hydrophobicity of CAB, resulting in a better performance of composite nanofibers [111]. PEG-based nano-hydroxyapatite and PEG-based nano-bioglass scaffolds showed positive results in physicochemical, mechanical, and cellular studies, which were attributed to the functional application of those materials in hard tissue engineering applications [112]. Polyvinyl alcohol (PVA), a water-soluble, non-toxic, biocompatible synthetic polymer, showed great potential as a biomaterial in wound-dressing applications [113]; the blended PVA with a biopolymer chitosan forms interconnected porous structure facilitated their functional use as a scaffold in soft-tissue engineering. [114]. The in vitro cytocompatibility of PVA and chitosan-based, double-network hydrogels was studied in rat bone marrow stem cells and mouse fibroblast cells, which revealed that the hydrogel was safe and non-toxic to the cells. The in vivo study displayed complete wound closure and no scar tissue formation in the hydrogel-treated groups, enhancing its use in tissue engineering applications [115]. PVA and gelatin-based hydrogel mimicked the suitable morphology and biological properties for tissue formation, which can be used as a cartilage scaffold for osteoarthritis surgery [116]. Some PVA-based scaffolds are successfully used in different tissue engineering applications, which include cardiovascular tissue engineering [117], bone tissue engineering [118], hard tissue regeneration [119], cartilage repair [120], and skin tissue regeneration [121].

## 3. The Potential Role of Nanocarriers in Tissue Engineering

Recently, the use of nanocarriers in tissue engineering applications has greatly increased due to their biological, mechanical, electrical, and antibacterial properties [122]. Nanocarriers are non-immunogenic in nature, non-toxic, highly biocompatible, biodegradable, provide mechanical strength and improve the hydrophilicity of scaffold material for tissue engineering applications [123].

The physicochemical properties of nanocarriers facilitate the easy and efficient loading of active biomolecules such as protein, enzyme, growth factor, drug, and natural medicinal compounds that are functionally applied in tissue engineering. The physicochemical properties of nanocarriers ensure the stability and nativity of the loaded biomolecules and facilitate controlled drug release mechanisms, which also play a key role in the degradation of the scaffold material. Nanocarriers often enhance the cytocompatibility of the implanted scaffold material and provide a suitable surface morphology to improve the different cellular functions such as cell viability, cell migration, cell attachment and cell proliferation.

A recent study suggested that the nanogel-based scaffolds play an effective role in tissue regeneration constructs for tissue engineering applications [124,125,126]. Pullulan-based nanogels were successfully synthesized and two different growth factors, such as human recombinant human bone morphogenetic protein 2 (BMP2) and recombinant human fibroblast growth factor 18 (FGF18), were loaded for effective bone repair in bone tissue engineering. The degradation of nanogels facilitated the sustained release of the growth factor and provided bone healing and regeneration in vivo [127]. The fibronectin-loaded nanogel-based 3D scaffolds showed positive outcomes in bone regeneration therapy. The biocompatibility and porous structure of the nanogels allowed the osteoblast cells to effectively adhere to the scaffold surface and contact to the loaded fibronectin [128].

**Figure 1:**
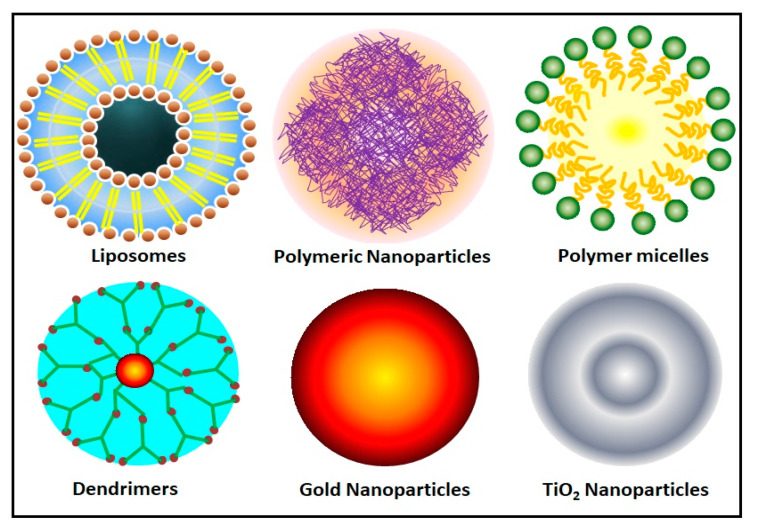
Schematic illustration of different types of nanocarrier used for tissue engineering applications.

**Figure 2:**
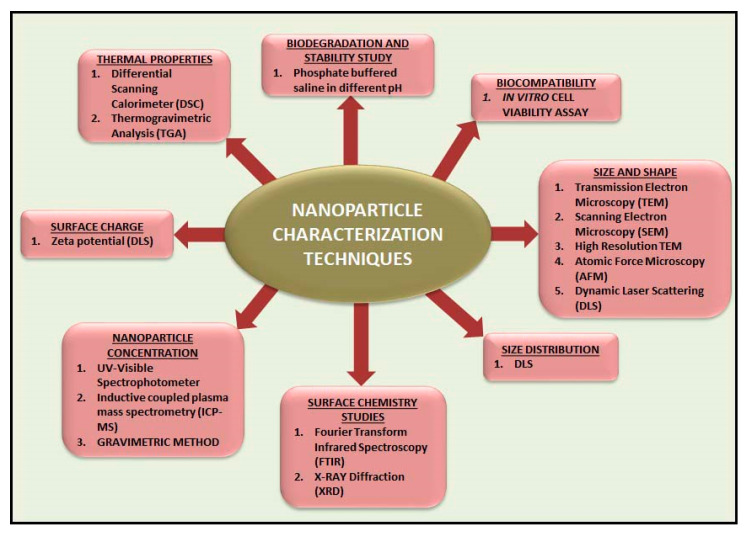
Different types of nanomaterial characterization techniques.

There are different types of nanocarriers are functionally used in tissue engineering applications, including polymeric nanoparticles [129], gold nanoparticles [130], titanium oxide nanoparticles [131], dendrimers [132], liposomes [133] and micelle [134]. A schematic illustration of the different types of nanocarrier used for tissue engineering applications is shown in Figure 1 and different types of nanomaterial characterization techniques are illustrated in Figure 2. The fabrication methodology, potential role, and application of nanocarrier-based biomaterials for tissue engineering applications are discussed in Table 1.

## 4. Effects of Natural Compounds Incorporated Scaffold in Tissue Engineering

**Figure 3:**
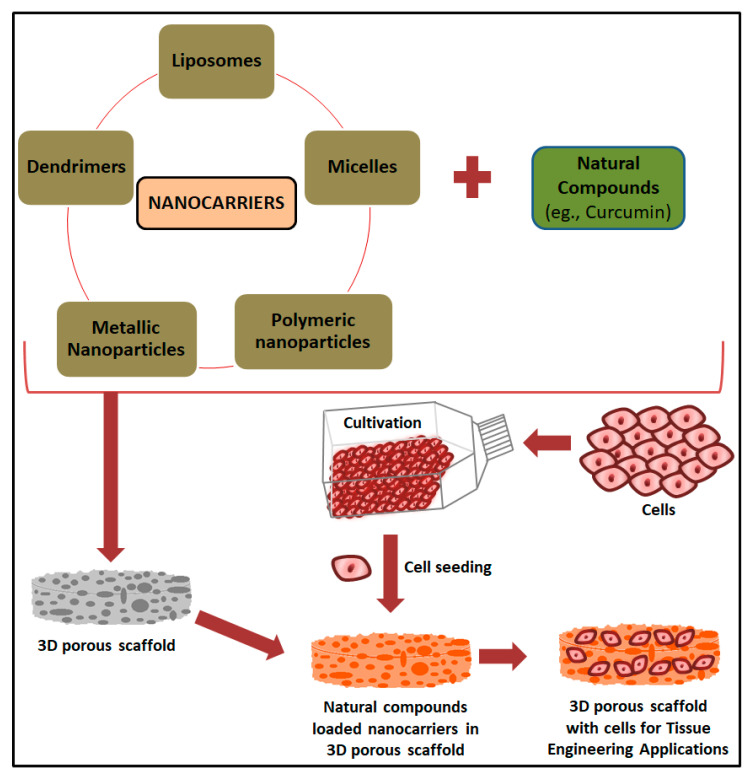
Nanocarriers and biomaterial-based scaffold for the delivery of natural medicinal compounds for tissue engineering applications.

**Figure 4:**
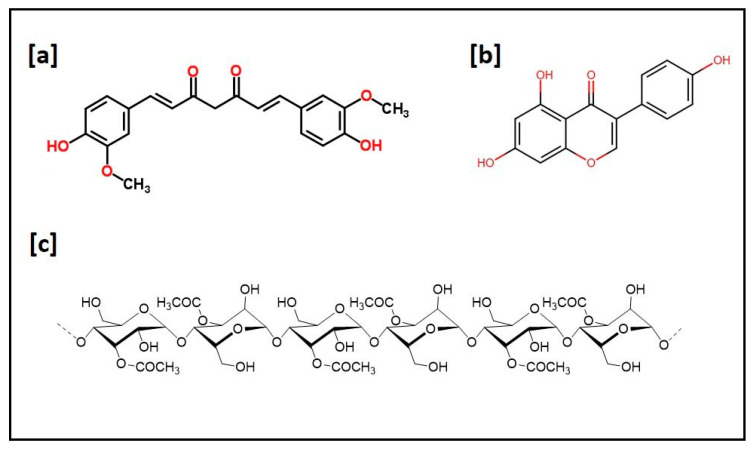
Chemical structure of natural compounds (**a**) curcumin; (**b**) soy isoflavones and (**c**) acemannan.

The use of natural compounds incorporated scaffolds shows a wide range of beneficial applications in tissue engineering. Bose et al. (2020) recently reported a comprehensive investigation on the osteogenic effects of natural medicinal compounds, the pharmacological effects of bioactive molecules present in the natural medicinal compounds and their potential role in bone tissue engineering [11]. A schematic representation of the preparation of a nanobiomaterial-based scaffold for the delivery of natural medicinal compounds for tissue engineering applications is shown in Figure 3 and the chemical structures of curcumin, soy isoflavones and acemannan are given in Figure 4. A comparative study on different types of natural compounds incorporating a scaffold for tissue engineering applications is given in Table 2. Turmeric (*Curcuma longa*), also known as “Indian saffron”, is traditionally used as a medicinal compound for different treatment modalities. Curcumin is considered to be one of the major bioactive constituents in turmeric and its therapeutic benefits are well documented in the literature [145,146]. It has wide application in tissue engineering and regenerative medicine due to its multifunctional biological activities, which include anti-inflammatory, anti-oxidant and antibacterial properties [147]. Although it has multifunctional activities and wide application in biological science, there are some issues associated with curcumin which limits its efficient use, such as low solubility, poor oral bioavailability, high degradation rate in alkaline pH, and photodegradation [146,148]. Nanotechnology platforms facilitates the bioavailability of curcumin by incorporation into different types of nanomaterials which include chitosan hydrogel [149], HA-PLA nanoparticles [150], gelatin-based biomimetic nanofibrous mats [151], SF nanoparticles [152], Pullulan-based nanoparticles [153], alginate-polysorbate 80 nanoparticles [154], PLA nanoparticles [155], PLGA nanoparticles [156], and polycaprolactone/montmorillonite nanocomposite [157].

The therapeutic properties of *Aloe vera* (Aloe barbadensis Miller) in wound-healing attracted the use of it as a biomaterial composite in tissue engineering applications [158]. *Aloe vera* plays an important role in the treatment of skin injury and has been used successfully in skin tissue engineering applications, improving the activity of amino acids, stimulating cell production, promoting skin regeneration, and preventing scar formation [159]. The bioactive compound “acemannan” is the major functional polysaccharide extracted from the leaves of *Aloe Sps.* It requires a deacetylation process using sodium borohydride to obtain the water-soluble form of acemannan and it showed greater antibacterial activity against both Gram-positive and Gram-negative bacterial strains [160]. It is commonly insoluble in acetone and propylene glycol and completely soluble in inorganic solvent (0.9% NaCl) [161]. Silva et al. (2013) explored the use of chitosan and *Aloe-vera* gel-based membranes as active wound dressing materials and showed desired physical, mechanical, and biological properties [162]. *Aloe vera* gel-based 3D sponges were prepared by freeze-drying techniques, and showed an interconnected pore structure in the matrix; gellan gum plays an important role in improving the stability and mechanical properties of sponges and can be used as an active biomaterial in regenerative medicine [163]. *Aloe vera* blended collagen-chitosan scaffolds facilitate cell migration and the porosity of the scaffold enhanced cellular activity in L3T3 mouse fibroblast cells [164]. *Aloe vera*-based Poly (vinyl pyrrolidone) (PVP) fibers were prepared by the electrospinning method, which has shown excellent antibacterial and antimicrobial activity against different microbial strains, and long-term storage studies found no microbial growth on the scaffold [165].

Soy protein extracted from soybean is inexpensive, has high storage capabilities, and has been identified as a rich source for more than 20 different amino acids, and soy-protein-based cellulose nanofibrils have played an important role in bone repair and regeneration of hard tissues [166]. Soy protein isolate (SPI) hydrolysates are more stable in heat than intact SPI, which leads to a greater solubility at pH 4.5 [167]. However, the film-forming ability of SPI was tested at different pH solutions, and it showed partially or totally denatured proteins at pH 11 and 2, but maintained their native conformation at pH 8. The structural differences in protein can affect the physical and mechanical properties of the films [168]. A wound-dressing material prepared using soy-protein-based bioactive glass nanoparticles was tested on mouse embryonic fibroblast cells and showed excellent cell viability and cytocompatibility [169]. In another study, soy-protein-based bioactive glass nanofibrous scaffolds showed a significant advantage in their use in tissue engineering [170]. A wide variety of soy-protein-loaded biomaterials have been developed and have had very positive effects on wound healing and applications in tissue engineering, including soy-protein-loaded, alginate hydrogel-based biomaterials [171], gentamicin-loaded, soy-protein-based matrix [172], and soy- and casein-based membrane [173].

## 5. Future Perspectives and Conclusions

In this review article, we discussed the functional application of natural medicinal compounds with incorporated biomaterials, and their potential role in various tissue engineering applications. Naturally derived compounds are biocompatible, non-toxic, biodegradable, and inexpensive in nature. The use of polymer-based materials reveal high benefits in the preparation of the biomaterial for tissue engineering applications, and this is well-documented in the literature. We believe that natural medicinal compounds with incorporated biomaterials have shown widespread biological and pharmacological effects in tissue engineering and regenerative medicine. A comprehensive literature study has shown that the latest advances in nanotechnology and their applications in tissue engineering have had positive effects on treatment modalities. Furthermore, the nano-based products facilitated the improved loading efficiency and sustained release of the natural medicinal compounds (e.g., curcumin), proteins, and growth factors, more efficiently, and the nanobiomaterials significantly overcame other limitations associated with those bioactive compounds. Plenty of bioactive compounds are available from natural sources; the unique clinical benefits of these compounds for tissue engineering applications need to be addressed in the future. The preparation of composite biomaterials using naturally available biopolymers with other types of polymers will overcome the interrelated side effects. The combination of polymers with inorganic and ceramic materials will improve the mechanical properties of the biomaterials. More research is needed to develop novel biomaterials using a combination of different polymers for tissue engineering applications. The development of novel biomaterials offers excellent opportunities in tissue engineering and regenerative medicine, and must meet the requirements of clinicians and comply with the expectations of patients. Furthermore, we recommend more in vivo and clinical trials to address the functional applications of natural medicinal-compound-loaded biomaterials for different tissue engineering applications.

## Figures and Tables

**Table 1:** Potential role and application of nanocarrier-based biomaterial for tissue engineering applications.

Name of the Nanocarrier Loaded Biomaterial/Composite/Scaffolds	Fabrication Techniques	Role of Nanocarriers	Tissue Engineering Applications	Outcomes
Nano Zinc Oxide (nZnO) and polycaprolactone based nanofiber.	Electrospinning method	Antibacterial properties	Bone tissue regeneration	The scaffold provides a nanoporous environment, which helped to increase cell adhesion and proliferation in MH63 cells [109].
Zeolite-nanoHAp based PCL/PLA nanofibers	Hydrothermal method (nanoHAp and Zeolite) and Electrospinning technique (nanofiber)	nanoHAp—bioactive ceramic in dentistry	Dental tissue regeneration	Plain PCL and PLA nanofibers showed low cell adhesion and migration due to their poor hydrophilic and smooth surface properties. Zeolite- and nHA-based composites overcome the limitations associated with PCL and PLA nanofibers and had positive outcomes on the osteoconductivity and osteoinductivity of scaffold for bone and tooth tissue engineering applications [129].
Gold nanoparticles loaded HAp and collagen-based biomaterial.	Chemical precipitation techniques—HAp nanomaterials. Microwave-assisted rapid heating methods—Gold loaded HAp	Carrier molecule	Tissue engineering	The synthesized biomaterials have shown excellent cytocompatibility against MG-63 osteoblast cells and been suitable as an ECM in tissue engineering. Gold loading concentration was considered an important parameter and it showed little toxicity when it reached 0.5% [130].
Nano TiO_2_ loaded SF-based nanocomposite	Freeze drying method	It leads mechanical interlocking and induces bone formation	Bone tissue engineering	High TiO_2_ concentrations (15 wt.%) improved the bioactivity behavior, and cell attachment. The low concentrations of TiO_2_ (5 wt.%) allowed the cells to spread only on the surface [131].
Dexamethasone-loaded carboxymethyl chitosan/poly(amidoamine) dendrimer nanoparticles	Precipitation method	Regulation of osteogenesis (in vivo)	Bone tissue engineering	An in vivo rat study showed that the synthesized dendrimer-based nanoparticles acted as an excellent intracellular nanocarrier for dexamethasone release and significantly enhanced the ectopic bone formation [132].
Paclitaxel-liposome loaded collagen microchannel scaffolds	Lyophilization method	The bilayer membrane of liposomes can help to improve the solubility issues associated with hydrophobic drugs such as paclitaxel.	Spinal cord injury repair	Sustained release of paclitaxel was achieved. It alleviates myelin inhibition and enhance neuronal differentiation (in vitro). It provides microenvironment support for neural stem cells to differentiate into mature neurons (in vivo) [133].
Nanofibrous micelles	Quenching, self-assembly and soft lithography approaches	It regulates cellular responses	Cellular alignment in tissue engineering	It mimics native fibrous networks surrounded by cells [134].
TiO_2_ Nanoparticles loaded porous PLGA-based scaffolds.	3D-printing technique	To improve mechanical properties of the scaffold	Bone tissue engineering	Osteoblast proliferation considerably increased in PLGA/TiO_2_ compared to pure PLGA [135].
Mesoporous silica nanoparticles (MSN) loaded collagen hydrogel.	Conventional method	Porous morphology to load nerve growth factor (NGF)	Neural tissue engineering	NGF-loaded collagen-MSN scaffolds show significant effects on neurite outgrowth patterns compared to NGF-loaded scaffold without MSN [136].
Nano-hydroxyapatite (HAp)-alginate-gelatin based microcapsule	Electrostatic encapsulation method	Nano-HAp promotes microencapsulated cell osteogenesis	Bone tissue engineering	The composite provided an efficient osteogenic building block. Alginate improves the swelling, stability, and mechanical strength of hydrogels. Further studies related to the composition of the hydrogels are required to improve their performance in static and dynamic cultures [137].
Nano-HAp, pullulan/dextran based composite	Freeze drying	Induced mineralization	Bone tissue engineering	The composite activates early calcification and osteoid tissue formation [138].
Nano silver, HAp, gelatin, alginate, poly (vinyl alcohol) based 3D scaffolds	Freezing thawing approach	Antibacterial activity	Bone tissue engineering	The 3D scaffold showed superior mechanical properties. The release of silver ions from scaffold materials leads to enhanced antibacterial activity against *Bacillus and E.coli sps.* Although it shows some positive outcomes in in vitro, more in vivo studies are required to find the suitability of the synthesized material for human beings [139].
Nano zirconia (nano ZrO_2_) loaded chitosan and SF-based nanocomposite	Freeze drying method	Chemical stability, mechanical and biocompatibility property for bone scaffolds	Tissue engineering	The interconnected porous composite material showed better physical, and mechanical properties. Enhanced biocompatibility and proliferation were observed in Human Gingival Fibroblast cells compared to the control [140].
Nano-HAp loaded polyhydroxybutyrate-co-(3-hydroxyvalerate) (PHBV) and SF-based composite.	Electrospinning methods	Nano-sized HAp promote cellular activity and rate of mineralization	Bone tissue engineering	The scaffold supports the attachment and proliferation of human osteoblast cells. The mechanical properties of this matrix show the decreased Young’s modulus when increasing concentration to 5 wt.% [141].
TiO_2_ Nanotube loaded 3D porous PLGA-based microspheres.	Single emulsion and microsphere sintered techniques	To provide compressive modulus and strength,	Bone tissue engineering	The existence of TiO_2_ improved the bioactivity of PLGA scaffold, promoting cell attachment (in vitro) and enhanced bone regeneration (in vivo) [142].
Mesoporous silica nanoparticles (MSNPs) loaded PLGA/gelatin nanofibrous scaffolds.	Electrospinning method for scaffold, Template removal method for MSNPs	To increase solution viscosity, conductivity, and hydrophilicity of the scaffolds	Nerve regeneration	The surface morphology, physical and biological properties of the scaffolds made it more suitable for nerve tissue engineering applications [143].
Strontium-doped HAp/SF biocomposite nanospheres	Ultrasonic coprecipitation method	Osteoinductive components	Bone regeneration	The synthesized nanospheres are biocompatible, facilitating osteogenic differentiation and osteoinductive properties (in vitro). The limitation of this study is that the author did not show the in situ bone defect healing potential of strontium-doped HAp/SF biocomposite nanospheres, but their hypothesis strongly recommended the use of this biomaterial as an in situ bone filling material [144].

**Table 2:** A comparative study on various types of natural compounds incorporated scaffold for tissue engineering application.

Detail of the Scaffold Material	Fabrication Type	Active Medicinal Compound Incorporated	Potential Role, Physicochemical Properties, and the Release Profile of Incorporated Active Medicinal Compound from the Nanobiomaterials	Outcomes
Novel Graphene oxide (GO) and Zn-Curcumin based composite nanofibers	Electrospinning	Curcumin	Core-shell nanofibers (153 nm diameter). Core (Zinc and curcumin complex) and shell (blend of carboxymethyl chitosan, PVA and GO) part of the nanofiber was confirmed through FTIR and XRD analysis. The presence of GO in the blend aided to improve the mechanical properties of nanofibers. In vitro drug release studies were performed for 25 days and revealed that the curcumin release was slower and more prolonged from nanofibers	The synthesized Zn-curcumin composite nanofibers showed excellent support for cell adhesion, spreading and the proliferation process and enhanced the activity of alkaline phosphatase. It has good antibacterial activity and promising potential for bone tissue engineering [174].
Composite nanofibrous scaffold (Curcumin incorporated chitosan, collagen, and polyvinyl-alcohol polymer-based nanofibers)	Electrospinning	Curcumin	The presence of nanometer sized fibers with interconnected pores were confirmed through scanning electron microscopy (SEM) study. An in vitro curcumin release from nanofibers was observed in phosphate-buffered saline (PBS) at 37 °C, which showed that the 20% of initial burst release in 24 h and sustained cumulative curcumin release was slowly increased by almost 90%, observed over a period of 21 days	A biocompatible scaffold used for tissue engineering applications, with well-interconnected pores helping to achieve optimal curcumin release, and increased cell attachment and cell viability. The nanofiber scaffold with curcumin showed higher α-SMA protein expression than the nanofiber scaffold without curcumin [175].
Bifunctional 3D printed scaffold (Liposome encapsulated curcumin onto 3D printed tricalcium phosphate (TCP))	Thin-film hydration	Curcumin	Transmission electron microscope (TEM) study revealed that the curcumin-encapsulated liposomes showed homogenous size distribution in the range of 40–50 nm. The properties of liposomes showed more controlled and sustained drug release of curcumin (17% released in 60 days)	It helps prevent bone cancer cells and promotes healthy bone cells, and this liposome-based, curcumin-loaded, bifunctional, 3D-printed scaffold can be used as a potential substitute to bone graft treatments after tumor removal [176].
3D printed biodegradable scaffolds (Curcumin, polyurethane, and gelatin)	One-step 3D printing process	Curcumin	The surface hydrophilicity, crosslinking density and nanoporous structure of the scaffold facilitated curcumin release. The burst release of curcumin was observed due to the surface hydrophilicity of the synthesized scaffolds.	Hydrophilic biodegradable porous scaffold exhibits excellent cell adhesion and cell proliferation properties. It can be used to regenerate cartilage tissues [177].
Biomimetic nanocomposite scaffolds (Polycaprolactone, Chitosan, Gelatin and Curcumin)	Freeze drying	Curcumin	SEM image revealed that the size of curcumin-loaded nanofibers was 139 nm, whereas the curcumin-free nanofibers were 195 nm. The addition of curcumin significantly reduced the size of the nanofibers. Slow curcumin release was observed in all types of scaffold studied in this work.	It mimics the ECM structure of soft tissues and showed suitable physicochemical and biological properties for skin regeneration [178].
SF-based biofunctional nanofibrous scaffold	Electrospinning method	*Aloe vera*	The field emission SEM study revealed the average size fiber diameter of *Aloe-vera*-loaded nanofiber was in the range of 212 ± 27 nm. The successful incorporation of aloe compound in the scaffold was confirmed through FTIR study.	The biological responses of the synthesized nanofibrous scaffolds, such as cell adhesion and migration, have been evaluated, and they provide a stable environment in the growth of human dermal fibroblasts for skin tissue engineering applications [179].
Polycaprolactone, chitosan and *Aloe vera* (AV) blended nanofiber membranes	Electrospinning method	*Aloe vera*	2% of AV plays an important role in the size of the nanofibers diameters, making it not easy to break. The average size diameter of nanofibers was 37.58 ± 3.24 (sloping free surface electrospinning method) and 53.63 ± 12.31 (modified bubble electrospinning method)	It has shown enhanced antibacterial activity against *E. coli and S. aureus* and Cytocompatibility against human umbilical vein endothelial cells. It is suiSection for treating acute wounds [180].
Alginate based hydrogel	Solvent-casting process	*Aloe vera*	The chemical composition of AV existence in the hydrogel was confirmed through FTIR study and thermogravimetric analysis results showed that the presence of AV increased the thermal stability of the material.	The synthesized films were evaluated with different physical and mechanical properties and could be applied for skin applications. The loading efficiency of *Aloe vera* was greatly increased due to the water absorption and swelling behavior of the hydrogel film [181].
Biodegradable soybean-based biomaterial	Thermosetting	Soybean	Genistein isoflavones from soybean could stimulate protein synthesis and osteoblastic functions and it plays a major role in bone regeneration (in vivo). The degradation of soybean granules was observed in the periphery of the defects through polarized light microscopy.	An in vivo rabbit study confirmed the osteogenic potential of the soybean-based biomaterial as a bone filler for bone regeneration [182].
Soybean-based biomaterial granules	Simple thermosetting method	Soybean	Genistein is one of the soy isoflavones present in the soybean. Approximately 0.08 µg/mL genistein release was observed after 100 h of the study in PBS pH 7.4 at 37 °C	An in vitro study has revealed that it reduced the activity of macrophages, differentiates osteoblast and may be functionally used for bone regeneration [183].
Multifunctional 3D printed TCP scaffolds	Binder jetting technique	Soy isoflavones	The multifunctional scaffold was prepared using all the three soy isoflavones in the ratio of 5:4:1 (genistein, daidzein and glycitein) and the release of all three isoflavones were observed in both pH 7.4 and 5.0 for 16 days. It revealed that 72.5% (genistein), 100% (daidzein) and 13.75% (glycitein) release in pH 7.4 and 25.1% (genistein), 23.3% (daidzein) and 2.97% (glycitein) release in acidic pH 5.0	It may be used in postsurgical applications, which include bone graft substitutes, drug delivery vehicle, localized tumor cell suppression and bone cell proliferation. The scaffolds must be tested with other malignant cell lines to confirm their chemopreventive efficacy and characterizations related to the expression of different bone markers [184].

## Abbreviations

2D	Two-dimensional
3D	Three-dimensional
ALP	Alkaline phosphatase
ECM	Extracellular matrix
FDA	Food and Drug Administration
G1 phase	Gap 1 phase cell cycle
HA	Hyaluronic acid
HAp	Hydroxyapatite
HEK293	Human Embryonic Kidney cells
HepG2	Hepatocellular carcinoma cells
hMSCs	Human mesenchymal stem cells
L-929	Fibroblast cells
MC3T3-E1	Pre-osteoblast cells
MG-63	Osteoblast like fibroblast cells
MSN or MSNPs	Mesoporous silica nanoparticles
NGF	Nerve growth factor
NIH3T3	Fibroblast cells
nZnO	Nano Zinc Oxide
PC12	Pheochromocytoma cells
PCL	Polycaprolactone
PGA	Polyglycolic acid
PHBV	Polyhydroxybutyrate-co-(3-hydroxyvalerate)
PLA	Polylactic acid
PLGA	Poly (lactic-co-glycolic acid)
PLL	Poly-L-lysine
PLLA	Poly (L-lactic acid)
RT-PCR	Real time—Polymerase chain reaction
Saos-2	Osteosarcoma epithelial cells
Sca-1	Stem cells antigen 1
SF	Silk fibroin
TCP	Tricalcium phosphate
TiO_2_	Titanium Oxide
VEGF	Vascular endothelial growth factor
WHO	World Health Organization
Zn	Zinc
ZrO_2_	Zirconia

## References

[1] Rezaei R., Safaeikatouli M., Mozaffari H.R., Moradpoor H., Karami S., Golshah A., Salimi B., Karami H. (2019). The Role of Nanomaterials in the Treatment of Diseases and Their Effects on the Immune System. Open Access Maced. J. Med. Sci..

[2] Patil M., Mehta D.S., Guvva S. (2008). Future impact of nanotechnology on medicine and dentistry. J. Indian Soc. Periodontol..

[3] Shi J., Votruba A.R., Farokhzad O.C., Langer R. (2010). Nanotechnology in Drug Delivery and Tissue Engineering: From Discovery to Applications. Nano Lett..

[4] Sahai N., Ahmad N., Gogoi M. (2018). Nanoparticles Based Drug Delivery for Tissue Regeneration Using Biodegradable Scaffolds: A Review. Curr. Pathobiol. Rep..

[5] Olson J.L., Atala A., Yoo J.J. (2011). Tissue Engineering: Current Strategies and Future Directions. Chonnam. Med. J..

[6] Eltom A., Zhong G., Muhammad A. (2019). Scaffold Techniques and Designs in Tissue Engineering Functions and Purposes: A Review. Adv. Mater. Sci. Eng..

[7] O’Brien F.J. (2011). Biomaterials & scaffolds for tissue engineering. Mater. Today.

[8] Chen F.-M., Liu X. (2016). Advancing biomaterials of human origin for tissue engineering. Prog. Polym. Sci..

[9] Patra J.K., Das G., Fraceto L.F., Campos E.V.R., Rodriguez-Torres M.D.P., Acosta-Torres L.S., Diaz-Torres L., Grillo R., Swamy M.K., Sharma S. (2018). Nano based drug delivery systems: Recent developments and future prospects. J. Nanobiotechnol..

[10] Mahomoodally M. (2013). Traditional Medicines in Africa: An Appraisal of Ten Potent African Medicinal Plants. Evid. Based Complement. Altern. Med..

[11] Bose S., Sarkar N. (2020). Natural Medicinal Compounds in Bone Tissue Engineering. Trends Biotechnol..

[12] Stratton S., Shelke N.B., Hoshino K., Rudraiah S., Kumbar S.G. (2016). Bioactive polymeric scaffolds for tissue engineering. Bioact. Mater..

[13] Abdulghani S., Mitchell G. (2019). Biomaterials for In Situ Tissue Regeneration: A Review. Biomolecules.

[14] Shao W., He J., Sang F., Ding B., Chen L., Cui S., Li K., Han Q., Tan W. (2016). Coaxial electrospun aligned tussah silk fibroin nanostructured fiber scaffolds embedded with hydroxyapatite–tussah silk fibroin nanoparticles for bone tissue engineering. Mater. Sci. Eng. C.

[15] Parenteau-Bareil R., Gauvin R., Berthod F. (2010). Collagen-Based Biomaterials for Tissue Engineering Applications. Materials.

[16] Wu W.-Q., Peng S., Song Z.-Y., Lin S. (2019). Collagen biomaterial for the treatment of myocardial infarction: An update on cardiac tissue engineering and myocardial regeneration. Drug Deliv. Transl. Res..

[17] Shi C., Li Q., Zhao Y., Chen W., Chen B., Xiao Z., Lin H., Nie L., Wang N., Dai J. (2011). Stem-cell-capturing collagen scaffold promotes cardiac tissue regeneration. Biomaterials.

[18] Yoon D., Cho Y.S., Joo S.Y., Seo C.H., Cho Y.-S. (2019). A clinical trial with a novel collagen dermal substitute for wound healing in burn patients. Biomater. Sci..

[19] Boccafoschi F., Habermehl J., Vesentini S., Mantovani D. (2005). Biological performances of collagen-based scaffolds for vascular tissue engineering. Biomaterials.

[20] Minardi S., Taraballi F., Wang X., Cabrera F.J., Van Eps J.L., Robbins A.B., Sandri M., Moreno M.R., Weiner B.K., Tasciotti E. (2017). Biomimetic collagen/elastin meshes for ventral hernia repair in a rat model. Acta Biomater..

[21] Kasoju N., Bora U. (2012). Silk Fibroin in Tissue Engineering. Adv. Healthc. Mater..

[22] Altman G.H., Diaz F., Jakuba C., Calabro T., Horan R.L., Chen J., Lu H., Richmond J., Kaplan D.L. (2003). Silk-based biomaterials. Biomaterials.

[23] Ma D., Wang Y., Dai W. (2018). Silk fibroin-based biomaterials for musculoskeletal tissue engineering. Mater. Sci. Eng. C.

[24] Melke J., Midha S., Ghosh S., Ito K., Hofmann S. (2016). Silk fibroin as biomaterial for bone tissue engineering. Acta Biomater..

[25] Wang Y., Wang X., Shi J., Zhu R., Zhang J., Zhang Z., Ma D., Hou Y., Lin F., Yang J. (2016). A Biomimetic Silk Fibroin/Sodium Alginate Composite Scaffold for Soft Tissue Engineering. Sci. Rep..

[26] Singh B.N., Pramanik K. (2018). Fabrication and evaluation of non-mulberry silk fibroin fiber reinforced chitosan based porous composite scaffold for cartilage tissue engineering. Tissue Cell.

[27] Yan C., Ren Y., Sun X., Jin L., Liu X., Chen H., Wang K., Yu M., Zhao Y. (2020). Photoluminescent functionalized carbon quantum dots loaded electroactive Silk fibroin/PLA nanofibrous bioactive scaffolds for cardiac tissue engineering. J. Photochem. Photobiol. B Biol..

[28] Boni R., Ali A., Shavandi A., Clarkson A.N. (2018). Current and novel polymeric biomaterials for neural tissue engineering. J. Biomed. Sci..

[29] Mallepally R.R., Marin M.A., Surampudi V., Subia B., Rao R.R., Kundu S.C., McHugh M.A. (2015). Silk fibroin aerogels: Potential scaffolds for tissue engineering applications. Biomed. Mater..

[30] Bhardwaj N., Nguyen Q.T., Chen A.C., Kaplan D.L., Sah R.L., Kundu S.C. (2011). Potential of 3-D tissue constructs engineered from bovine chondrocytes/silk fibroin-chitosan for in vitro cartilage tissue engineering. Biomaterials.

[31] Mobika J., Rajkumar M., Priya V.N., Sibi S.L. (2020). Substantial effect of silk fibroin reinforcement on properties of hydroxyapatite/silk fibroin nanocomposite for bone tissue engineering application. J. Mol. Struct..

[32] Reizabal A., Brito-Pereira R., Fernandes M.M., Castro N., Correia V., Ribeiro C., Costa C.M., Perez L., Vilas J., Lanceros-Méndez S. (2020). Silk fibroin magnetoactive nanocomposite films and membranes for dynamic bone tissue engineering strategies. Materials.

[33] Wöltje M., Böbel M., Bienert M., Neuss S., Aibibu D., Cherif C. (2018). Functionalized silk fibers from transgenic silkworms for wound healing applications: Surface presentation of bioactive epidermal growth factor. J. Biomed. Mater. Res. Part A.

[34] Echave M.C., Burgo L.S., Pedraz J.L., Orive G., Echave M.C. (2017). Gelatin as Biomaterial for Tissue Engineering. Curr. Pharm. Des..

[35] Li Z., Qu T., Ding C., Ma C., Sun H., Li S., Liu X. (2014). Injectable gelatin derivative hydrogels with sustained vascular endothelial growth factor release for induced angiogenesis. Acta Biomater..

[36] Bello A.B., Kim D., Kim D., Park H., Lee S.-H. (2020). Engineering and Functionalization of Gelatin Biomaterials: From Cell Culture to Medical Applications. Tissue Eng. Part B Rev..

[37] Marino A., Tonda-Turo C., De Pasquale D., Ruini F., Genchi G., Nitti S., Cappello V., Gemmi M., Mattoli V., Ciardelli G. (2017). Gelatin/nanoceria nanocomposite fibers as antioxidant scaffolds for neuronal regeneration. Biochim. Biophys. Acta (BBA) Gen. Subj..

[38] Ali M.G., Mousa H.M., Blaudez F., El-Sadek M.A., Mohamed M., Abdel-Jaber G., Abdal-Hay A., Ivanovski S. (2020). Dual nanofiber scaffolds composed of polyurethane-gelatin/nylon 6- gelatin for bone tissue engineering. Colloids Surf. A Physicochem. Eng. Asp..

[39] Nooeaid P., Chuysinuan P., Pengsuk C., Dechtrirat D., Lirdprapamongkol K., Techasakul S., Svasti J. (2020). Polylactic Acid Microparticles Embedded Porous Gelatin Scaffolds with Multifunctional properties for Soft Tissue Engineering. J. Sci. Adv. Mater. Devices.

[40] Sharifi F., Irani S., Azadegan G., Pezeshki-Modaress M., Zandi M., Saeed M. (2020). Co-electrospun gelatin-chondroitin sulfate/polycaprolactone nanofibrous scaffolds for cartilage tissue engineering. Bioact. Carbohydr. Diet. Fibre.

[41] Govindan R., Gu F., Karthi S., Girija E. (2020). Effect of phosphate glass reinforcement on the mechanical and biological properties of freeze-dried gelatin composite scaffolds for bone tissue engineering applications. Mater. Today Commun..

[42] Rouse J.G., Van Dyke M.E. (2010). A Review of Keratin-Based Biomaterials for Biomedical Applications. Materials.

[43] Lu T.-Y., Huang W.-C., Chen Y., Baskaran N., Yu J., Wei Y., Kumar B.N. (2020). Effect of Varied Hair Protein Fractions on the Gel Properties of Keratin/Chitosan Hydrogels for the Use in Tissue Engineering. Colloids Surf. B Biointerfaces.

[44] Balaji S., Kumar R., Sripriya R., Kakkar P., Ramesh D.V., Reddy P.N.K., Sehgal P. (2012). Preparation and comparative characterization of keratin–chitosan and keratin-gelatin composite scaffolds for tissue engineering applications. Mater. Sci. Eng. C.

[45] Lv X., Li Z., Zhang M., Xie M., Huang J., Peng X., Yang R., Wang H., Xu Y.-M., Feng C. (2016). Structural and functional evaluation of oxygenating keratin/silk fibroin scaffold and initial assessment of their potential for urethral tissue engineering. Biomaterials.

[46] Dou J., Wang Y., Jin X., Li P., Wang L., Yuan J., Shen J. (2020). PCL/sulfonated keratin mats for vascular tissue engineering scaffold with potential of catalytic nitric oxide generation. Mater. Sci. Eng. C.

[47] Muzzarelli R.A. (2011). Chitosan composites with inorganics, morphogenetic proteins and stem cells, for bone regeneration. Carbohydr. Polym..

[48] Rodríguez-Vázquez M., Vega-Ruiz B., Ramos-Zúñiga R., Saldaña-Koppel D.A., Quiñones-Olvera L.F. (2015). Chitosan and Its Potential Use as a Scaffold for Tissue Engineering in Regenerative Medicine. BioMed Res. Int..

[49] Kiuchi H., Kai W., Inoue Y. (2007). Preparation and characterization of poly(ethylene glycol) crosslinked chitosan films. J. Appl. Polym. Sci..

[50] Gümüşderelioğlu M., Aday S. (2011). Heparin-functionalized chitosan scaffolds for bone tissue engineering. Carbohydr. Res..

[51] Li G., Xiao Q., Zhang L., Zhao Y., Yang Y. (2017). Nerve growth factor loaded heparin/chitosan scaffolds for accelerating peripheral nerve regeneration. Carbohydr. Polym..

[52] Du F., Wang H., Zhao W., Li N., Kong D., Yang J., Zhang Y. (2012). Gradient nanofibrous chitosan/poly ε-caprolactone scaffolds as extracellular microenvironments for vascular tissue engineering. Biomaterials.

[53] Gomes S., Rodrigues G., Martins G.G., Henriques C., Silva J.C. (2017). Evaluation of nanofibrous scaffolds obtained from blends of chitosan, gelatin and polycaprolactone for skin tissue engineering. Int. J. Biol. Macromol..

[54] Wang S., Sun C., Guan S., Li W., Xu J., Ge D., Zhuang M., Liu T., Ma X. (2017). Chitosan/gelatin porous scaffolds assembled with conductive poly(3,4-ethylenedioxythiophene) nanoparticles for neural tissue engineering. J. Mater. Chem. B.

[55] Yan L., Wang Y.-J., Ren L., Wu G., Caridade S.G., Fan J.-B., Wang L.-Y., Ji P.-H., Oliveira J., Oliveira J.T. (2010). Genipin-cross-linked collagen/chitosan biomimetic scaffolds for articular cartilage tissue engineering applications. J. Biomed. Mater. Res. Part A.

[56] Aya K.L., Stern R. (2014). Hyaluronan in wound healing: Rediscovering a major player. Wound Repair Regen..

[57] Spicer C.D. (2020). Hydrogel scaffolds for tissue engineering: The importance of polymer choice. Polym. Chem..

[58] Li F., Ducker M., Sun B., Szele F., Czernuszka J.T. (2020). Interpenetrating polymer networks of collagen, hyaluronic acid, and chondroitin sulfate as scaffolds for brain tissue engineering. Acta Biomater..

[59] Movahedi M., Asefnejad A., Rafienia M., Khorasani M.T. (2020). Potential of novel electrospun core-shell structured polyurethane/starch (hyaluronic acid) nanofibers for skin tissue engineering: In vitro and in vivo evaluation. Int. J. Biol. Macromol..

[60] Suner S.S., Demirci S., Yetiskin B., Fakhrullin R., Naumenko E., Okay O., Ayyala R.S., Sahiner N. (2019). Cryogel composites based on hyaluronic acid and halloysite nanotubes as scaffold for tissue engineering. Int. J. Biol. Macromol..

[61] Kenar H., Ozdogan C.Y., Dumlu C., Doger E., Kose G.T., Hasirci V. (2019). Microfibrous scaffolds from poly(l-lactide-co-ε-caprolactone) blended with xeno-free collagen/hyaluronic acid for improvement of vascularization in tissue engineering applications. Mater. Sci. Eng. C.

[62] Florczyk S.J., Wang K., Jana S., Wood D.L., Sytsma S.K., Sham J.G., Kievit F.M., Zhang M. (2013). Porous chitosan-hyaluronic acid scaffolds as a mimic of glioblastoma microenvironment ECM. Biomaterials.

[63] Correia C.R., Moreira-Teixeira L.S., Moroni L., Reis R.L., van Blitterswijk C.A., Karperien M., Mano J.F. (2011). Chitosan scaffolds containing hyaluronic acid for cartilage tissue engineering. Tissue Eng. Part C Methods.

[64] Tan H., Chu C.R., Payne K.A., Marra K.G. (2009). Injectable in situ forming biodegradable chitosan–hyaluronic acid based hydrogels for cartilage tissue engineering. Biomaterials.

[65] Coimbra P., Alves P., Valente T., Santos R., Correia I.J., Ferreira P. (2011). Sodium hyaluronate/chitosan polyelectrolyte complex scaffolds for dental pulp regeneration: Synthesis and characterization. Int. J. Biol. Macromol..

[66] Lee E.J., Kang E., Kang S.-W., Huh K.M. (2020). Thermo-irreversible glycol chitosan/hyaluronic acid blend hydrogel for injectable tissue engineering. Carbohydr. Polym..

[67] Unnithan A.R., Sasikala A.R.K., Kim C.S., Kim C.S. (2017). A unique scaffold for bone tissue engineering: An osteogenic combination of graphene oxide–hyaluronic acid–chitosan with simvastatin. J. Ind. Eng. Chem..

[68] Lee S.J., Nah H., Heo D.N., Kim K.-H., Seok J.M., Heo M., Moon H.-J., Lee D., Lee J.S., An S.Y. (2020). Induction of osteogenic differentiation in a rat calvarial bone defect model using an In situ forming graphene oxide incorporated glycol chitosan/oxidized hyaluronic acid injectable hydrogel. Carbon.

[69] Gilarska A., Lewandowska-Lancucka J., Guzdek-Zajac K., Karewicz A., Horak W., Lach R., Wójcik K., Nowakowska M. (2020). Bioactive yet antimicrobial structurally stable collagen/chitosan/lysine functionalized hyaluronic acid-based injectable hydrogels for potential bone tissue engineering applications. Int. J. Biol. Macromol..

[70] Singh R.S., Kaur N., Rana V., Kennedy J.F. (2016). Recent insights on applications of pullulan in tissue engineering. Carbohydr. Polym..

[71] Bae H., Ahari A.F., Shin H., Nichol J.W., Hutson C.B., Masaeli M., Kim S.-H., Aubin H., Yamanlar S., Khademhosseini A. (2011). Cell-laden microengineered pullulan methacrylate hydrogels promote cell proliferation and 3D cluster formation. Soft Matter.

[72] Arora A., Sharma P., Katti D.S. (2015). Pullulan-based composite scaffolds for bone tissue engineering: Improved osteoconductivity by pore wall mineralization. Carbohydr. Polym..

[73] Han Y., Lv S. (2019). Synthesis of chemically crosslinked pullulan/gelatin-based extracellular matrix-mimetic gels. Int. J. Biol. Macromol..

[74] Dalgic A.D., Atila D., Karatas A., Tezcaner A., Keskin D. (2019). Diatom shell incorporated PHBV/PCL-pullulan co-electrospun scaffold for bone tissue engineering. Mater. Sci. Eng. C.

[75] Schlaubitz S., Derkaoui S.M., Marosa L., Miraux S., Renard M., Catros S., Le Visage C., Letourneur D., Amédée J., Fricain J.-C. (2014). Pullulan/dextran/nHA Macroporous Composite Beads for Bone Repair in a Femoral Condyle Defect in Rats. PLoS ONE.

[76] Della Giustina G., Gandin A., Brigo L., Panciera T., Giulitti S., Sgarbossa P., D’Alessandro D., Trombi L., Danti S., Brusatin G. (2019). Polysaccharide hydrogels for multiscale 3D printing of pullulan scaffolds. Mater. Des..

[77] Ghorbani F., Zamanian A., Behnamghader A., Joupari M.D. (2020). Bioactive and biostable hyaluronic acid-pullulan dermal hydrogels incorporated with biomimetic hydroxyapatite spheres. Mater. Sci. Eng. C.

[78] Atila D., Keskin D., Tezcaner A. (2016). Crosslinked pullulan/cellulose acetate fibrous scaffolds for bone tissue engineering. Mater. Sci. Eng. C.

[79] Yang J., Shen M., Wen H., Luo Y., Huang R., Rong L., Xie J. (2019). Recent advance in delivery system and tissue engineering applications of chondroitin sulfate. Carbohydr. Polym..

[80] Kwon H.J., Han Y. (2016). Chondroitin sulfate-based biomaterials for tissue engineering. Turk. J. Biol..

[81] Sadeghi A., Zandi M., Pezeshki-Modaress M., Rajabi S. (2019). Tough, hybrid chondroitin sulfate nanofibers as a promising scaffold for skin tissue engineering. Int. J. Biol. Macromol..

[82] Venkatesan J., Pallela R., Bhatnagar I., Kim S.-K. (2012). Chitosan-amylopectin/hydroxyapatite and chitosan-chondroitin sulphate/hydroxyapatite composite scaffolds for bone tissue engineering. Int. J. Biol. Macromol..

[83] Piai J.F., Da Silva M.L.A., Martins A., Torres A.B., Faria S., Reis R.L., Muniz E.C., Neves N. (2017). Chondroitin sulfate immobilization at the surface of electrospun nanofiber meshes for cartilage tissue regeneration approaches. Appl. Surf. Sci..

[84] Zhou F., Zhang X., Cai D., Li J., Mu Q., Zhang W., Zhu S., Jiang Y.Z., Shen W.L., Zhang S. (2017). Silk fibroin-chondroitin sulfate scaffold with immuno-inhibition property for articular cartilage repair. Acta Biomater..

[85] Bhowmick S., Rother S., Zimmermann H., Lee P.S., Moeller S., Schnabelrauch M., Koul V., Jordan R., Hintze V., Scharnweber D. (2017). Biomimetic electrospun scaffolds from main extracellular matrix components for skin tissue engineering application—The role of chondroitin sulfate and sulfated hyaluronan. Mater. Sci. Eng. C.

[86] Saporito F., Sandri G., Bonferoni M., Rossi S., Malavasi L., Del Fante C., Vigani B., Black L.D., Ferrari F. (2018). Electrospun Gelatin–Chondroitin Sulfate Scaffolds Loaded with Platelet Lysate Promote Immature Cardiomyocyte Proliferation. Polymers.

[87] Zărnescu O., Craciunescu O., Seciu A.-M., Stanciuc A.M., Moldovan L. (2018). Collagen-chondroitin 4-sulfate-fibronectin scaffold: Characterization and in vitro biocompatibility. J. Biotechnol..

[88] Yang J., Jiang S., Guan Y., Deng J., Lou S., Feng D., Kong D., Li C. (2019). Pancreatic islet surface engineering with a starPEG-chondroitin sulfate nanocoating. Biomater. Sci..

[89] Singh B.N., Veeresh V., Mallick S.P., Jain Y., Sinha S., Rastogi A., Srivastava P. (2019). Design and evaluation of chitosan/chondroitin sulfate/nano-bioglass based composite scaffold for bone tissue engineering. Int. J. Biol. Macromol..

[90] Fenbo M., Sijing L., Ruiz-Ortega L., Yuanjun Z., Lei X., Kui W., Lijun L., Bin T. (2020). Effects of alginate/chondroitin sulfate-based hydrogels on bone defects healing. Mater. Sci. Eng. C.

[91] Ma F.B., Xia X.Y., Tang B. (2019). Strontium chondroitin sulfate/silk fibroin blend membrane containing microporous structure modulates macrophage responses for guided bone regeneration. Carbohydr. Polym..

[92] Kudryavtseva V., Stankevich K.S., Gudima A., Kibler E., Zvereva I.A., Bolbasov E., Malashicheva A., Zhuravlev M., Riabov V., Liu T. (2017). Atmospheric pressure plasma assisted immobilization of hyaluronic acid on tissue engineering PLA-based scaffolds and its effect on primary human macrophages. Mater. Des..

[93] Hassanajili S., Pour A.K., Oryan A., Talaei-Khozani T. (2019). Preparation and characterization of PLA/PCL/HA composite scaffolds using indirect 3D printing for bone tissue engineering. Mater. Sci. Eng. C.

[94] Oryan A., Hassanajili S., Sahvieh S., Azarpira N. (2020). Effectiveness of mesenchymal stem cell-seeded onto the 3D polylactic acid/polycaprolactone/hydroxyapatite scaffold on the radius bone defect in rat. Life Sci..

[95] Zhang L.-K., Wang H., Yang R., Liu M., Ban Q., Chen W., Zhao M., You R., Jin Y., Guan Y.-Q. (2019). Bone marrow stem cells combined with polycaprolactone-polylactic acid-polypropylene amine scaffolds for the treatment of acute liver failure. Chem. Eng. J..

[96] Gentile P., Chiono V., Carmagnola I., Hatton P. (2014). An Overview of Poly(lactic-co-glycolic) Acid (PLGA)-Based Biomaterials for Bone Tissue Engineering. Int. J. Mol. Sci..

[97] Kerimoglu O., Alarcin E. (2012). Poly(Lactic-Co-Glycolic Acid) Based Drug Delivery Devices For Tissue Engineering And Regenerative Medicine. ANKEM Derg..

[98] Nokhasteh S., Sadeghi-Avalshahr A., Molavi A.M., Khorsand-Ghayeni M., Naderi-Meshkin H., Molavi A.M. (2018). Effect of bioactive glass nanoparticles on biological properties of PLGA/collagen scaffold. Prog. Biomater..

[99] Mooney D.J., Mazzoni C.L., Breuer C., McNamara K., Hern D., Vacanti J.P., Langer R. (1996). Stabilized polyglycolic acid fibre-based tubes for tissue engineering. Biomaterials.

[100] Zhang J., Yang S., Yang X., Xi Z., Zhao L., Cen L., Lu E., Yang Y. (2018). Novel Fabricating Process for Porous Polyglycolic Acid Scaffolds by Melt-Foaming Using Supercritical Carbon Dioxide. ACS Biomater. Sci. Eng..

[101] Yuan Y., Shi X., Gan Z., Wang F. (2018). Modification of porous PLGA microspheres by poly-l-lysine for use as tissue engineering scaffolds. Colloids Surf. B Biointerfaces.

[102] Ong Y.X.J., Lee L.Y., Davoodi P., Wang C.-H. (2018). Production of drug-releasing biodegradable microporous scaffold using a two-step micro-encapsulation/supercritical foaming process. J. Supercrit. Fluids.

[103] Amirthalingam M., Kasinathan N., Amuthan A., Mutalik S., Reddy M.S., Nayanabhirama U. (2016). Bioactive PLGA–curcumin microparticle-embedded chitosan scaffold: In vitro and in vivo evaluation. Artif. Cells Nanomed. Biotechnol..

[104] Wu S., Zhou R., Zhou F., Streubel P.N., Chen S., Duan B. (2019). Electrospun Thymosin Beta-4 Loaded PLGA/PLA Nanofiber/Microfiber Hybrid Yarns for Tendon Tissue Engineering Application. Mater. Sci. Eng. C.

[105] Qodratnama R., Serino L.P., Cox H.C., Qutachi O., White L.J. (2015). Formulations for modulation of protein release from large-size PLGA microparticles for tissue engineering. Mater. Sci. Eng. C.

[106] Khojasteh A., Fahimipour F., Eslaminejad M.B., Jafarian M., Jahangir S., Bastami F., Tahriri M., Karkhaneh A., Tayebi L. (2016). Development of PLGA-coated β-TCP scaffolds containing VEGF for bone tissue engineering. Mater. Sci. Eng. C.

[107] Fahimipour F., Rasoulianboroujeni M., Dashtimoghadam E., Khoshroo K., Tahriri M., Bastami F., Lobner D., Tayebi L. (2017). 3D printed TCP-based scaffold incorporating VEGF-loaded PLGA microspheres for craniofacial tissue engineering. Dent. Mater..

[108] Dwivedi R., Kumar S., Pandey R., Mahajan A., Nandana D., Katti D.S., Mehrotra D. (2020). Polycaprolactone as biomaterial for bone scaffolds: Review of literature. J. Oral Biol. Craniofacial Res..

[109] Harikrishnan P., Sivasamy A. (2020). Preparation, characterization of Electrospun Polycaprolactone-nano Zinc oxide composite scaffolds for Osteogenic applications. Nano Struct. Nano Objects.

[110] Neufurth M., Wang X., Wang S., Steffen R., Ackermann M., Haep N.D., Schröder H.C., Müller W.E. (2017). 3D printing of hybrid biomaterials for bone tissue engineering: Calcium-polyphosphate microparticles encapsulated by polycaprolactone. Acta Biomater..

[111] Tan H.-L., Kai D., Pasbakhsh P., Teow S.-Y., Lim Y.-Y., Pushpamalar J. (2020). Electrospun cellulose acetate butyrate/polyethylene glycol (CAB/PEG) composite nanofibers: A potential scaffold for tissue engineering. Colloids Surf. B Biointerfaces.

[112] Kumar P., Dehiya B.S., Sindhu A. (2019). Synthesis and characterization of nHA-PEG and nBG-PEG scaffolds for hard tissue engineering applications. Ceram. Int..

[113] Kamoun E.A., Kenawy E.-R., Tamer T.M., El-Meligy M.A., Eldin M.S.M. (2015). Poly(vinyl alcohol)-alginate physically crosslinked hydrogel membranes for wound dressing applications: Characterization and bio-evaluation. Arab. J. Chem..

[114] Kanimozhi K., Basha S.K., Kumari V.S. (2016). Fabrication of chitosan based hybrid porous scaffolds by salt leaching for soft tissue engineering. Surf. Interfaces.

[115] Bi S., Pang J., Huang L., Sun M., Cheng X.J., Chen X. (2020). The toughness chitosan-PVA double network hydrogel based on alkali solution system and hydrogen bonding for tissue engineering applications. Int. J. Biol. Macromol..

[116] Thangprasert A., Tansakul C., Thuaksubun N., Meesane J. (2019). Mimicked hybrid hydrogel based on gelatin/PVA for tissue engineering in subchondral bone interface for osteoarthritis surgery. Mater. Des..

[117] Mombini S., Mohammadnejad J., Bakhshandeh B., Narmani A., Nourmohammadi J., Vahdat S., Zirak S. (2019). Chitosan-PVA-CNT nanofibers as electrically conductive scaffolds for cardiovascular tissue engineering. Int. J. Biol. Macromol..

[118] Ali A., Bano S., Priyadarshi R., Negi Y.S. (2019). Effect of carbon based fillers on properties of Chitosan/PVA/βTCP based composite scaffold for bone tissue engineering. Mater. Today Proc..

[119] Kim H., Yang G.H., Choi C.H., Cho Y.-S., Kim G.H. (2018). Gelatin/PVA scaffolds fabricated using a 3D-printing process employed with a low-temperature plate for hard tissue regeneration: Fabrication and characterizations. Int. J. Biol. Macromol..

[120] Lin B., Hu H., Deng Z., Pang L., Jiang H., Wang D., Li J., Liu Z., Wang H., Zeng X. (2020). Novel bioactive glass cross-linked PVA hydrogel with enhanced chondrogenesis properties and application in mice chondrocytes for cartilage repair. J. Non-Cryst. Solids.

[121] Koosha M., Raoufi M., Moravvej H. (2019). One-pot reactive electrospinning of chitosan/PVA hydrogel nanofibers reinforced by halloysite nanotubes with enhanced fibroblast cell attachment for skin tissue regeneration. Colloids Surf. B Biointerfaces.

[122] Hasan A., Morshed M., Memic A., Hassan S., Webster T.J., Marei H.E. (2018). Nanoparticles in tissue engineering: Applications, challenges and prospects. Int. J. Nanomed..

[123] Eftekhari A., Dizaj S.M., Sharifi S., Salatin S., Saadat Y.R., Vahed S.Z., Samiei M., Ardalan M., Rameshrad M., Ahmadian E. (2020). The Use of Nanomaterials in Tissue Engineering for Cartilage Regeneration; Current Approaches and Future Perspectives. Int. J. Mol. Sci..

[124] Ghaeini-Hesaroeiye S., Bagtash H.R.R., Boddohi S., Vasheghani-F E., Jabbari E. (2020). Thermoresponsive Nanogels Based on Different Polymeric Moieties for Biomedical Applications. Gels.

[125] Vicario-De-La-Torre M., Forcada J. (2017). The Potential of Stimuli-Responsive Nanogels in Drug and Active Molecule Delivery for Targeted Therapy. Gels.

[126] Grimaudo M.A., Concheiro A., Alvarez-Lorenzo C. (2019). Nanogels for regenerative medicine. J. Control. Release.

[127] Fujioka-Kobayashi M., Ota M., Shimoda A., Nakahama K.-I., Akiyoshi K., Miyamoto Y., Iseki S. (2012). Cholesteryl group- and acryloyl group-bearing pullulan nanogel to deliver BMP2 and FGF18 for bone tissue engineering. Biomaterials.

[128] Sato Y., Yamamoto K., Horiguchi S., Tahara Y., Nakai K., Kotani S.-I., Oseko F., Pezzotti G., Yamamoto T., Kishida T. (2018). Nanogel tectonic porous 3D scaffold for direct reprogramming fibroblasts into osteoblasts and bone regeneration. Sci. Rep..

[129] Mohandesnezhad S., Pilehvar-Soltanahmadi Y., Alizadeh E., Goodarzi A., Davaran S., Khatamian M., Zarghami N., Samiei M., Aghazadeh M., Akbarzadeh A. (2020). In vitro evaluation of Zeolite-nHA blended PCL/PLA nanofibers for dental tissue engineering. Mater. Chem. Phys..

[130] Mondal S., Hoang G., Manivasagan P., Moorthy M.S., Phan T.T.V., Kim H.H., Nguyen T.P., Oh J. (2019). Rapid microwave-assisted synthesis of gold loaded hydroxyapatite collagen nano-bio materials for drug delivery and tissue engineering application. Ceram. Int..

[131] Johari N., Hosseini H.M., Samadikuchaksaraei A. (2017). Optimized composition of nanocomposite scaffolds formed from silk fibroin and nano-TiO2 for bone tissue engineering. Mater. Sci. Eng. C.

[132] Oliveira J., Kotobuki N., Tadokoro M., Hirose M., Mano J.F., Reis R.L., Ohgushi H. (2010). Ex vivo culturing of stromal cells with dexamethasone-loaded carboxymethylchitosan/poly(amidoamine) dendrimer nanoparticles promotes ectopic bone formation. Bone.

[133] Li X., Fan C., Xiao Z., Zhao Y., Zhang H., Sun J., Zhuang Y., Wu X., Shi J., Chen Y. (2018). A collagen microchannel scaffold carrying paclitaxel-liposomes induces neuronal differentiation of neural stem cells through Wnt/β-catenin signaling for spinal cord injury repair. Biomaterials.

[134] Zhang K., Arranja A., Chen H., Mytnyk S., Wang Y., Oldenhof S., Van Esch J.H., Mendes E. (2018). A nano-fibrous platform of copolymer patterned surfaces for controlled cell alignment. RSC Adv..

[135] Rasoulianboroujeni M., Fahimipour F., Shah P., Khoshroo K., Tahriri M., Eslami H., Yadegari A., Dashtimoghadam E., Tayebi L. (2019). Development of 3D-printed PLGA/TiO_2_ nanocomposite scaffolds for bone tissue engineering applications. Mater. Sci. Eng. C.

[136] Lee J.H., Park J.-H., Eltohamy M., Perez R.A., Lee E.-J., Kim H.-W. (2013). Collagen gel combined with mesoporous nanoparticles loading nerve growth factor as a feasible therapeutic three-dimensional depot for neural tissue engineering. RSC Adv..

[137] Nabavinia M., Khoshfetrat A.B., Naderi-Meshkin H. (2019). Nano-hydroxyapatite-alginate-gelatin microcapsule as a potential osteogenic building block for modular bone tissue engineering. Mater. Sci. Eng. C.

[138] Fricain J.C., Schlaubitz S., Visage C.L., Arnault I. (2013). A nano-hydroxyapatite—Pullulan/dextran polysaccharide composite macroporous material for bone tissue engineering. Biomaterials.

[139] Saini R.K., Bagri L.P., Bajpai A. (2019). Nano-silver hydroxyapatite based antibacterial 3D scaffolds of gelatin/alginate/poly (vinyl alcohol) for bone tissue engineering applications. Colloids Surf. B Biointerfaces.

[140] Teimour A., Ebrahimi R., Emadi R., Beni B.H., Chermahini A.N. (2015). Nano-composite of silk fibroin–chitosan/Nano ZrO_2_ for tissue engineering applications: Fabrication and morphology. Int. J. Biol. Macromol..

[141] Paşcu E.I., Stokes J., McGuinness G. (2013). Electrospun composites of PHBV, silk fibroin and nano-hydroxyapatite for bone tissue engineering. Mater. Sci. Eng. C.

[142] Eslami H., Lisar H.A., Kashi T.S.J., Tahriri M., Ansari M., Rafiei T., Bastami F., Shahin-Shamsabadi A., Abbas F.M., Tayebi L. (2018). Poly(lactic-co-glycolic acid)(PLGA)/TiO_2_ nanotube bioactive composite as a novel scaffold for bone tissue engineering: In vitro and in vivo studies. Biologicals.

[143] Mehrasa M., Asadollahi M.A., Ghaedi K., Salehi H., Arpanaei A. (2015). Electrospun aligned PLGA and PLGA/gelatin nanofibers embedded with silica nanoparticles for tissue engineering. Int. J. Biol. Macromol..

[144] Wang L., Pathak J.L., Liang D., Zhong N., Guan H., Wan M., Miao G., Li Z., Ge L. (2020). Fabrication and characterization of strontium-hydroxyapatite/silk fibroin biocomposite nanospheres for bone-tissue engineering applications. Int. J. Biol. Macromol..

[145] Kumar S.S.D., Houreld N.N., Abrahamse H. (2018). Therapeutic Potential and Recent Advances of Curcumin in the Treatment of Aging-Associated Diseases. Molecules.

[146] Kumar S.S.D., Mahadevan S., Vijayaraghavan R., Mandal A.B., MacFarlane D.R. (2014). Cucrumin loaded poly(2-hydroxymethyl methacrylate) nanoparticles from gelled ionic liquid—In vitro cytotoxicity and anti-cancer activity in SKOV-3 cells. Eur. J. Pharm. Sci..

[147] Ahangari N., Kargozar S., Ghayour-Mobarhan M., Baino F., Pasdar A., Sahebkar A., Ferns G.A.A., Kim H.-W., Mozafari M. (2018). Curcumin in tissue engineering: A traditional remedy for modern medicine. BioFactors.

[148] Kumar S.S.D., Mahesh A., Mahadevan S., Mandal A.B. (2014). Synthesis and characterization of curcumin loaded polymer/lipid based nanoparticles and evaluation of their antitumor effects on MCF-7 cells. Biochim. Biophys. Acta (BBA) Gen. Subj..

[149] Gunathilake T.M.S.U., Ching Y.C., Chuah C.H. (2017). Enhancement of Curcumin Bioavailability Using Nanocellulose Reinforced Chitosan Hydrogel. Polymers.

[150] Chen Y.-N., Hsu S.-L., Liao M.-Y., Liu Y.-T., Lai C.-H., Chen J.-F., Nguyen M.-H.T., Su Y.-H., Chen S.-T., Wu L.-C. (2016). Ameliorative Effect of Curcumin-Encapsulated Hyaluronic Acid–PLA Nanoparticles on Thioacetamide-Induced Murine Hepatic Fibrosis. Int. J. Environ. Res. Public Health.

[151] Dai X., Liu J., Zheng H., Wichmann J., Hopfner U., Sudhop S., Prein C., Shen Y., Machens H.-G., Schilling A.F. (2017). Nano-formulated curcumin accelerates acute wound healing through Dkk-1-mediated fibroblast mobilization and MCP-1-mediated anti-inflammation. NPG Asia Mater..

[152] Montalban M.G., Coburn J.M., Lozano-Pérez A.A., Cenis J.L., Víllora G., Kaplan D.L. (2018). Production of Curcumin-Loaded Silk Fibroin Nanoparticles for Cancer Therapy. Nanomaterials.

[153] Ganeshkumar M., Ponrasu T., Subamekala M.K., Janani M., Suguna L. (2016). Curcumin loaded on pullulan acetate nanoparticles protects the liver from damage induced by DEN. RSC Adv..

[154] Govindaraju R., Karki R., Chandrashekarappa J., Santhanam M., Shankar A.K., Joshi H.K., Divakar G., Roopa G., Jayanthi C., Mukunthan S. (2019). Enhanced Water Dispersibility of Curcumin Encapsulated in Alginate-polysorbate 80 Nano Particles and Bioavailability in Healthy Human Volunteers. Pharm. Nanotechnol..

[155] Rachmawati H., Yanda Y.L., Rahma A., Mase N. (2016). Curcumin-Loaded PLA Nanoparticles: Formulation and Physical Evaluation. Sci. Pharm..

[156] Anand P., Nair H.B.B., Sung B., Kunnumakkara A.B., Yadav V.R., Tekmal R.R., Aggarwal B.B. (2010). RETRACTED: Design of curcumin-loaded PLGA nanoparticles formulation with enhanced cellular uptake, and increased bioactivity in vitro and superior bioavailability in vivo. Biochem. Pharmacol..

[157] Sadeghianmaryan A., Yazdanpanah Z., Soltani Y.A., Sardroud H.A., Nasirtabrizi M.H., Chen X., Nasirtabrizi M.H. (2019). Curcumin-loaded electrospun polycaprolactone/montmorillonite nanocomposite: Wound dressing application with anti-bacterial and low cell toxicity properties. J. Biomater. Sci. Polym. Ed..

[158] Maan A.A., Nazir A., Khan M.K.I., Ahmad T., Zia R., Murid M., Abrar M. (2018). The therapeutic properties and applications of Aloe vera: A review. J. Herb. Med..

[159] Eshun K., He Q. (2004). Aloe Vera: A Valuable Ingredient for the Food, Pharmaceutical and Cosmetic Industries—A Review. Crit. Rev. Food Sci. Nutr..

[160] Salah F., El-Ghoul Y., Mahdhi A., Majdoub H., Jarroux N., Sakli F. (2017). Effect of the deacetylation degree on the antibacterial and antibiofilm activity of acemannan from Aloe vera. Ind. Crops Prod..

[161] Nema J., Shrivastava S.K., Mitra N.G. (2012). Physicochemical study of acemannan polysaccharide in Aloe species under the influence of soil reaction (pH) and moisture application. Afr. J. Pure Appl. Chem..

[162] Silva S.S., Popa E.G., Gomes M.E., Cerqueira M.T., Marques A.P., Caridade S.G., Teixeira P., Sousa C., Mano J.F., Reis R.L. (2013). An investigation of the potential application of chitosan/aloe-based membranes for regenerative medicine. Acta Biomater..

[163] Silva S.S., Oliveira M.B., Mano J.F., Reis R.L. (2014). Bio-inspired Aloe vera sponges for biomedical applications. Carbohydr. Polym..

[164] Jithendra P., Rajam A.M., Kalaivani T., Mandal A.B., Rose C. (2013). Preparation and Characterization of Aloe Vera Blended Collagen-Chitosan Composite Scaffold for Tissue Engineering Applications. ACS Appl. Mater. Interfaces.

[165] Aghamohamadi N., Sharifi-Sanjani N., Majidi R.F., Nasrollahi S.A. (2018). Preparation and characterization of Aloe vera acetate and electrospinning fibers as promising antibacterial properties materials. Mater. Sci. Eng. C.

[166] Salama A., Abou-Zeid R.E., Cruz-Maya I., Guarino V. (2019). Soy protein hydrolysate grafted cellulose nanofibrils with bioactive signals for bone repair and regeneration. Carbohydr. Polym..

[167] Kim S.Y., Park P.S.W., Rhee K.C. (1990). Functional properties of proteolytic enzyme modified soy protein isolate. J. Agric. Food Chem..

[168] Mauri A.N., Añón M.C. (2006). Effect of solution pH on solubility and some structural properties of soybean protein isolate films. J. Sci. Food Agric..

[169] Tansaz S., Schulte M., Kneser U., Mohn D., Stark W., Roether J.A., Cicha I., Boccaccini A.R. (2018). Soy protein isolate/bioactive glass composite membranes: Processing and properties. Eur. Polym. J..

[170] Tansaz S., Liverani L., Vester L., Boccaccini A.R. (2017). Soy protein meets bioactive glass: Electrospun composite fibers for tissue engineering applications. Mater. Lett..

[171] Tansaz S., Durmann A.-K., Detsch R., Boccaccini A.R. (2016). Hydrogel films and microcapsules based on soy protein isolate combined with alginate. J. Appl. Polym. Sci..

[172] Peles Z., Zilberman M. (2012). Novel soy protein wound dressings with controlled antibiotic release: Mechanical and physical properties. Acta Biomater..

[173] Silva G., Vaz C.M., Coutinho O.P., Cunha A.M., Reis R.L. (2003). In vitro degradation and cytocompatibility evaluation of novel soy and sodium caseinate-based membrane biomaterials. J. Mater. Sci. Mater. Electron..

[174] Sedghi R., Sayyari N., Shaabani A., Niknejad H., Tayebi T. (2018). Novel biocompatible zinc-curcumin loaded coaxial nanofibers for bone tissue engineering application. Polymers.

[175] Mokhames Z., Rezaie Z., Ardeshirylajimi A., Basiri A., Taheri M., Omrani M.D. (2020). Efficient smooth muscle cell differentiation of iPS cells on curcumin-incorporated chitosan/collagen/polyvinyl-alcohol nanofibers. In Vitro Cell. Dev. Biol. Anim..

[176] Sarkar N., Bose S. (2019). Liposome-Encapsulated Curcumin-Loaded 3D Printed Scaffold for Bone Tissue Engineering. ACS Appl. Mater. Interfaces.

[177] Lee M.J., Kim S.E., Park J., Ahn G.Y., Yun T.H., Choi I., Kim H., Choi S.-W. (2019). Curcumin-loaded biodegradable polyurethane scaffolds modified with gelatin using 3D printing technology for cartilage tissue engineering. Polym. Adv. Technol..

[178] Ghaee A., Bagheri-Khoulenjani S., Afshar H.A., Bogheiri H. (2019). Biomimetic nanocomposite scaffolds based on surface modified PCL-nanofibers containing curcumin embedded in chitosan/gelatin for skin regeneration. Compos. Part B Eng..

[179] Suganya S., Venugopal J., Ramakrishna S., Lakshmi B., Dev V.R.G. (2014). Naturally derived biofunctional nanofibrous scaffold for skin tissue regeneration. Int. J. Biol. Macromol..

[180] Yin J., Xu L. (2020). Batch preparation of electrospun polycaprolactone/chitosan/aloe vera blended nanofiber membranes for novel wound dressing. Int. J. Biol. Macromol..

[181] Pereira R.F.B., Carvalho A., Vaz D.C., Gil M.H., Mendes A., Bartolo P.J. (2013). Development of novel alginate based hydrogel films for wound healing applications. Int. J. Biol. Macromol..

[182] Merolli A., Nicolais L., Ambrosio L., Santin M. (2010). A degradable soybean-based biomaterial used effectively as a bone filler in vivo in a rabbit. Biomed. Mater..

[183] Santin M., Morris C., Standen G., Nicolais L., Ambrosio L. (2007). A New Class of Bioactive and Biodegradable Soybean-Based Bone Fillers. Biomacromolecules.

[184] Sarkar N., Bose S. (2020). Controlled release of soy isoflavones from multifunctional 3D printed bone tissue engineering scaffolds. Acta Biomater..

